# Effect of tumour necrotic factor‐α, interleukin‐17 and interleukin‐22 on the expression of filaggerin‐2 and hornerin: Analysis of a three‐dimensional psoriatic skin model

**DOI:** 10.1002/ski2.440

**Published:** 2024-09-05

**Authors:** Teruhiko Makino, Megumi Mizawa, Keita Takemoto, Seiji Yamamoto, Tadamichi Shimizu

**Affiliations:** ^1^ Department of Dermatology Faculty of Medicine Academic Assembly University of Toyama Toyama Japan; ^2^ Department of Pathology Faculty of Medicine Academic Assembly University of Toyama Toyama Japan

## Abstract

**Background:**

Filaggrin‐2 (FLG2) and hornerin (HRNR) are members of the S100 fused‐type protein family, which share many properties with filaggrin (FLG). A previous study demonstrated that the expression of FLG2 was significantly decreased in psoriatic skin relative to that in normal skin. In contrast, the HRNR expression in psoriatic skin varied among studies.

**Objectives:**

We aimed to investigate the effect of tumour necrosis factor‐α (TNF‐α), IL‐17A and IL‐22 on the expression of FLG2 and HRNR using a three‐dimensional psoriatic skin model.

**Methods:**

In the present study, we generated a 3D psoriatic skin model that was stimulated with TNF‐α, IL‐17A and IL‐22 for 3D skin equivalents. Using this model, we examined the altered expression of FLG2 and HRNR by quantitative reverse transcription polymerase chain reaction and immunostaining.

**Results:**

In the 3D psoriatic skin model, the expression of FLG2 and HRNR was significantly reduced compared with that in the 3D control skin. FLG2 expression was significantly decreased by stimulation with TNF‐α, IL‐17A and IL‐22. In contrast, the expression of HRNR was markedly increased by stimulation with IL‐22, although it was slightly decreased by TNF‐α or IL‐17A compared to the 3D control skin.

**Conclusion:**

TNF‐α, IL‐17A and IL‐22 have different effects on FLG2 and HRNR expression and epidermal structure formation. Furthermore, FLG2 and HRNR may play different roles in barrier formation and the pathogenesis of psoriasis.

## Graphical abstract

We generated a three‐dimensional (3D) psoriatic skin model that was stimulated with TNF‐α, IL‐17A and IL‐22 for 3D skin equivalents. We used this model to examine the altered expression of FLG2 and HRNR. Consequently, FLG2 and HRNR may play different roles in barrier formation and the pathogenesis of psoriasis.

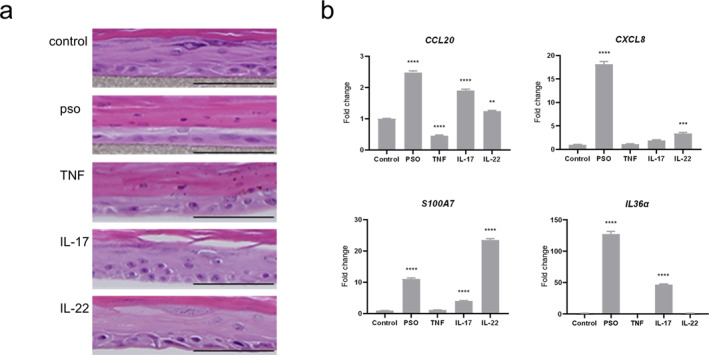

**What is already known about this topic?**

- FLG2 and HRNR are members of the S100 fused‐type protein family.
- Tumour necrosis factor‐α (TNF‐α), IL‐23p19 and the IL‐17A play fundamental roles in pathogenesis of psoriasis.
- The FLG expression was significantly reduced in psoriatic lesions.

**What does this study add?**

- Stimulation with TNF‐α, IL‐17A or IL‐22 significantly decreased the expressions of FLG2.
- Stimulation with IL‐22 significantly increased the expression of HRNR.
- TNF‐α, IL‐17A and IL‐22 may be involved, in a complex manner, in the expression of FLG2 and HRNR in the psoriatic skin.

## 1 INTRODUCTION

Recent progress in biological therapies for psoriasis has revealed the fundamental roles of tumour necrosis factor‐α (TNF‐α), interleukin (IL)‐23p19 and IL‐17A axis in pathogenesis. An association with interferon (IFN)‐α, IFN‐γ, IL‐12 and IL‐22 has also been reported.^1^ The S100 fused‐type protein family members (cornulin, filaggrin [FLG], filaggrin‐2 [FLG2], hornerin [HRNR], repetin, trichohyalin and trichohyalin‐like 1) are located at chromosome 1q21.3.^2^ Among of them, FLG2 and HRNR share many properties with FLG, including a closely related structural organization, a similar amino acid composition and an analogous proteolytic processing.^3^, ^4^ HRNR and FLG2 were therefore suggested to play a role in the epidermal barrier formation, the same as FLG.^3^, ^4^ Previous studies reported that the FLG expression was significantly reduced in psoriatic lesions.^5^, ^6^ We previously reported the altered expression of S100 fused‐type proteins in a three‐dimensional (3D) atopic dermatitis skin model, which was generated by stimulation with recombinant IL‐4 and IL‐13 for 3D skin equivalents.^7^ This 3D model was useful for assessing the direct effect of cytokines on the formation of the epidermal structure and the expression of differentiation‐associated proteins. Therefore, we examined the effects of TNF‐α, IL‐17A and IL‐22 on the expression of FLG2 and HRNR using a 3D psoriatic skin model to clarify the role of FLG2 and HRNR in psoriatic skin.

## 2 MATERIALS AND METHODS

### 2.1 Preparation of 3D skin disease models

To generate a 3D psoriatic skin model, EpiDerm was purchased from MatTek Corporation. Recombinant human TNF‐α (50 ng/μL: R&D Systems), IL‐17A (50 ng/μL: R&D Systems) and IL‐22 (50 ng/μL: R&D Systems) were added to the medium (EPI‐100: MatTek Corporation) and cultured for 5 days according to a previous study.^8^ Three‐dimensional skin models were independently generated in triplicate.

### 2.2 Quantitative reverse transcription polymerase chain reaction (RT‐PCR)

Total RNA was isolated from the 3D skin models using an RNeasy Mini Kit (QIAGEN) and treated with DNase 1 (QIAGEN). Reverse transcription was performed using Superscript III (ThermoFisher Scientific). Complementary DNA samples were analysed using SYBR Premix Ex Taq™ II (Takara Bio Inc.) according to the manufacturer's instructions. All experiments were performed in triplicate and the *β*‐actin levels were normalised. The primers used are listed in Table 1.

**TABLE 1:** Primers for quantitative RT‐PCR.

FLG	FW: GGAATTTCGGCAAATCCTG
	RV: GCTTGAGCCAACTTGAATACCA
FLG2	FW: GCACACTGAGCAAGGGTGAACTAA
	RV: AGGACCTTGTTGCAGGCCATA
HRNR	FW: ACGTTGAACAAGGCAGAGCTGA
	RV: CTCGATCCAGACTTTGCAAGATGA
CCL20	FW: AAAGTTGTCTGTGTGCGCAAATC
	RV: TTGGGCTATGTCCAATTCCATTC
CXCL8	FW: AGACAGCAGAGCACACAAGCTTCTA
	RV: GGCCAGCTTGGAAGTCATGTTTA
IL36α	FW: GGACTCAATCTCTGCCTGATGTG
	RV: ACAGGCTCGGGTTGGTTGTA
S100A7	FW: CACCAGACGTGATGACAAGATTGA
	RV: AGACATCGGCGAGGTAATTTGTG
ACTB	FW: TGGCACCCAGCACAATGAA
	RV: CTAAGTCATAGTCCGCCTAGAAGCA

Abbreviation: RT‐PCR, reverse transcription polymerase chain reaction.

### 2.3 Immunostaining

Immunostaining was performed as previously described.^3^ Antibodies against FLG were purchased from Abcam. Antibodies against FLG2 and HRNR were generated as previously described.^3^, ^4^ All experiments were independently repeated three times.

### 2.4 Statistical analysis

Values are expressed as mean ± standard deviation (SD). Statistical significance was evaluated using the Mann‐Whitney *U* test. Statistical significance was set at *p* < 0.05.

## 3 RESULTS

**FIGURE 1:**
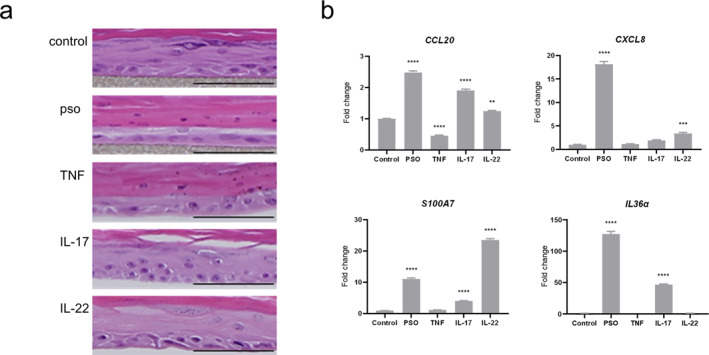
Histological findings and the cytokine expression in the 3D psoriatic skin model. (a) Histological findings of the 3D skin models. Scale bar: 100 μm (haematoxylin and eosin staining, original magnification 400×). (b) Relative mRNAs of CCL20, CXCL8, S100A7 and IL‐36α in the 3D skin models were each analysed by quantitative RT‐PCR and normalized to the *β*‐actin value. All data represent the mean ± SD of three independent experiments. *p < 0.05; **p < 0.01; ***p < 0.001; ****p < 0.0001 versus 3D control skin models. Control: 3D control skin model, pso: 3D psoriatic skin model, TNF: 3D TNF‐α skin model, IL‐17: 3D IL‐17A skin model, IL‐22: 3D IL‐22 skin model for all experiments. RT‐PCR, reverse transcription polymerase chain reaction.

We generated a 3D psoriatic skin model in which the 3D skin equivalent was stimulated by TNF‐α, IL‐17A and IL‐22. We also generated 3D skin equivalents stimulated with TNF‐α, IL‐17A or IL‐22 and named them 3D TNF‐α, 3D IL17A and 3D IL22 skin models, respectively. Histological analysis of the 3D psoriatic skin models showed hypoplastic epidermis, no keratohyalin granules and parakeratosis (Figure 1a). The histological findings of the 3D TNF‐α skin model were similar to those of the 3D psoriatic skin model. In contrast, the 3D IL17A and 3D IL22 skin models showed a hyperplastic epidermis in comparison to the 3D control skin model (Figure 1a). To confirm whether these models have the characteristics of a psoriatic epidermis, we examined the expression of CCL20 (a chemotactic factor of Th17 cells), CXCL8 (a neutrophil chemotactic factor), S100A7 (an antimicrobial peptide) and IL36α (a feedforward cytokine that amplifies the IL‐23/Il‐17 axis).^7^ All of which were significantly increased in the 3D psoriatic skin model, similar to the psoriatic epidermis (Figure 1b). Interestingly, the expressions of CCL20, CXCL8, S100A7 and IL36α were significantly induced by IL‐17A and IL‐22 (Figure 1b).

**FIGURE 2:**
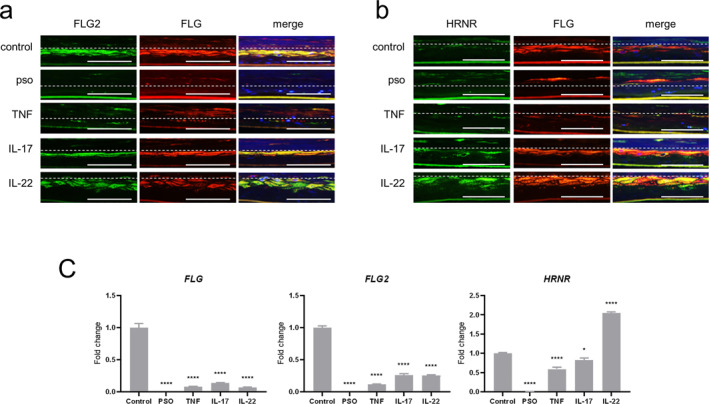
T altered expression of FLG‐2 and HRNR in the 3D psoriatic skin model. (a) 3D skin models were double‐stained with anti‐FLG2 and anti‐FLG antibodies. The dotted line indicates the boundary between the granular layer and the stratum corneum. Scale bar: 100 μm (original magnification 400×). (b) 3D skin models were stained with anti‐HRNR and anti‐FLG antibodies. The dotted line indicates the boundary between the granular layer and the stratum corneum. Scale bar: 100 μm (original magnification 400×). (c) Relative mRNAs of FLG, FLG2 and HRNR in 3D skin models were analysed by quantitative RT‐PCR and normalized to the *β*‐actin value. All data represent the mean ± SD of three independent experiments. *p < 0.05; **p < 0.01; ***p < 0.001; ****p < 0.0001 versus 3D control skin models. Control: 3D control skin model, pso: 3D psoriatic skin model: TNF: 3D TNF‐α skin model, IL‐17: 3D IL‐17A skin model, IL‐22: 3D IL‐22 skin model for all experiments. RT‐PCR, reverse transcription polymerase chain reaction.

Using these skin models, we examined FLG2 and HRNR expression. Immunohistochemistry revealed markedly decreased expression of FLG2 and HRNR in the granular layers of the 3D psoriatic skin model compared to the 3D control skin model (Figure 2a,b). Similarly, FLG2 and HRNR mRNAs expression was rarely observed in the 3D psoriatic skin model (Figure 2c). FLG2 immunohistochemically showed a marked decrease in the 3D TNF‐α skin model and a slight decrease in the 3D IL17A and IL22 skin models in comparison to 3D control skin model (Figure 2a); however, the expression of FLG2 mRNA in the 3D TNF‐α, IL17A and IL22 skin models was suppressed to 12.0%, 26.3% and 25.4% of the 3D control skin model, respectively (Figure 2c, centre panel). The FLG mRNA expression levels in these skin models were suppressed by 7.06%, 14.0% and 7.15%, respectively, relative to the 3D control skin model (Figure 2c, left panel). In contrast, immunohistochemistry revealed increased expression of HRNR in the granular layers of the 3D IL17A and IL22 skin models, relative to the 3D control skin model, while it was rarely observed in the 3D TNF‐α skin model (Figure 2b). HRNR signals were detected in some FLG‐expressing cells, whereas FLG2 and FLG signals were detected in almost the same cells in these 3D skin models (Figure 2a,b). HRNR mRNA expression levels in the 3D TNF‐α, IL17A and IL22 skin models were 59.0%, 82.5% and 204.7% of the 3D control skin model, respectively.

## 4 DISCUSSION

In the present study, we demonstrated the histological and biological characteristics of a 3D psoriatic skin model. These findings were similar to those of skin lesions in psoriasis, especially in parakeratotic area.^9^ In addition, the 3D TNFF‐α, IL17A and IL22 skin models clearly demonstrated the roles of TNF‐α, IL‐17A and IL‐22 in the formation of the epidermal structure and expression of psoriasis‐related molecules. Therefore, these 3D skin models were used in this study.

A previous study demonstrated that the expression of FLG2 was significantly decreased in psoriatic skin relative to that in normal skin.^3^ The FLG2 expression pattern in the 3D psoriatic skin model corresponded to that in psoriatic skin. Furthermore, FLG2 expression was significantly suppressed by stimulation with TNF‐α, IL‐17A and IL‐22, in parallel with FLG expression. The regulation of FLG2 expression may be similar to that of FLG expression and FLG2 may function in epidermal barrier formation in coordination with FLG.

HRNR is also known to be associated with epidermal cornification; however, the HRNR expression pattern appears to be unique. A previous report demonstrated the increased expression of HRNR in hyperproliferative epidermis (e.g., regenerating skin on wounds), although HRNR was rarely detected in normal skin.^4^ In addition, the increased expression of HRNR was observed in FLG‐knockdown 3D skin equivalent.^7^ In psoriatic skin, the HRNR expression varied among studies. Wu et al. demonstrated decreased expression of HRNR in psoriatic skin.^10^ In contrast, in our previous study, HRNR expression was induced in the hypergranulotic area of psoriatic skin but not in the parakeratotic area. The HRNR expression in the 3D psoriatic skin model may reflect that in the parakeratotic area of the psoriatic skin. Interestingly, the expression of HRNR was significantly decreased in the 3D TNF‐α skin model; however, it was significantly increased in the 3D IL22 skin model.

Although the mechanisms underlying HRNR regulation by TNF‐α, IL17A and IL22 remains unclear, a previous study demonstrated that IL22 upregulated the expression of keratin 17 via activation of signal transducer and activator of transcription 3 (STAT3) and extracellular signal‐regulated kinase 1/2 (ERK1/2).^11^ Keratin 17 is a hyperproliferation‐associated keratin, similar to keratin 6 and keratin 16, and is overexpressed in wound healing and in psoriatic skin lesions.^12^, ^13^ Because of the similarity of the expression patterns of HRNR and keratin 17, we speculate that signal pathways of STAT3 and ERK1/2 may be associated with the IL22‐induced expression of HRNR and that HRNR may be regulated by a different system from FLG and FLG2.

In conclusion, TNF‐α, IL‐17A and IL‐22 have different effects on FLG2 and HRNR expression and epidermal structure formation. In a complex manner, these cytokines may be involved in the expression of FLG2 and HRNR and the pathogenesis of psoriasis. This study demonstrated that FLG2 and HRNR may play different roles in barrier formation and the pathogenesis of psoriasis.

One limitation of the present study is that the results were based on the analysis of in vitro models. Therefore, future studies are needed to verify whether the findings observed in the present study are comparable in vivo and to clarify the underlying mechanisms of the cytokine‐mediated regulation of FLG2 and HRNR.

## CONFLICT OF INTEREST STATEMENT

None to declare.

## AUTHOR CONTRIBUTIONS

**Teruhiko Makino**: Conceptualization (lead); data curation (equal); formal analysis (lead); funding acquisition (lead); investigation (lead); methodology (lead); project administration (lead); validation (lead); visualization (lead); writing—original draft (lead); writing—review and editing (lead). **Megumi Mizawa**: Conceptualization (equal); data curation (equal); formal analysis (equal); investigation (equal); methodology (equal); project administration (equal); supervision (equal); validation (lead); visualization (equal); writing—original draft (equal); writing—review and editing (equal). **Keita Takemoto**: Conceptualization (supporting); data curation (supporting); formal analysis (equal); investigation (equal); methodology (supporting); project administration (equal); validation (equal); visualization (equal); writing—original draft (supporting); writing—review and editing (equal). **Seiji Yamamoto**: Conceptualization (equal); data curation (equal); formal analysis (lead); funding acquisition (lead); investigation (lead); methodology (lead); project administration (equal); validation (equal); visualization (lead); writing—original draft (equal); writing—review and editing (equal). **Tadamichi Shimizu**: Conceptualization (lead); data curation (equal); formal analysis (equal); investigation (lead); methodology (equal); project administration (lead); supervision (lead); validation (equal); visualization (equal); writing—original draft (equal); writing—review and editing (lead).

## ETHICS STATEMENT

Not applicable.

## PATIENT CONSENT

Not applicable.

## Data Availability

The datasets generated and/or analysed during the current study are available from the corresponding author on reasonable request.
